# Raising awareness of antimicrobial resistance in rural aquaculture practice in Bangladesh through digital communications: a pilot study

**DOI:** 10.1080/16549716.2020.1734735

**Published:** 2020-03-10

**Authors:** Kelly Thornber, Doina Huso, Muhammad Meezanur Rahman, Himangsu Biswas, Mohammad Habibur Rahman, Eric Brum, Charles R. Tyler

**Affiliations:** a Centre for Sustainable Aquaculture Futures, University of Exeter, Exeter, UK; b Biosciences, University of Exeter, Exeter, UK; c WorldFish Headquarters, Bayan Lepas, Penang, Malaysia; d WorldFish Bangladesh, World Fish Bangladesh Office, Banani, Dhaka, Bangladesh; e Emergency Centre for Transboundary Animal Diseases (ECTAD), Food and Agriculture Organization of the United Nations, Dhaka, Bangladesh

**Keywords:** Antibiotic, fish farming, South East Asia, low-income countries, animation, social media, awareness campaign

## Abstract

One of the key strategic objectives of the World Health Organisation’s global antimicrobial resistance (AMR) action plan is to improve public awareness and understanding of this issue. Very few AMR awareness campaigns have targeted the animal production sector, particularly in low- and middle-income countries (LMICs) where rural communities can be geographically difficult to access via traditional face-to-face community engagement methods. Aquaculture is a major food production industry in Bangladesh and across Asia, an area which poses a significant risk to global AMR dissemination. In this pilot study, we sought to investigate the potential for digital communication materials to rapidly and effectively communicate AMR messages to rural aquaculture farmers in Bangladesh. Working with stakeholders from the Bangladesh aquaculture industry, we developed a 4-minute digital animation designed specifically for this audience and assessed its capacity to engage and communicate AMR messages to farmers. We then conducted a small-scale social media campaign, to determine the potential for rapidly disseminating AMR awareness materials to a large audience across Bangladesh, where there is an extensive 4 G internet network and an ever-increasing proportion of the population (57% as of December 2019) have mobile internet access. Thirty-six farmers were surveyed: all of them liked this method of communication and 97% said it would change the way they use antibiotics in the future. Through the social media campaign, the animation received 9,100 views in the first 2 weeks alone. Although preliminary, these results demonstrate the huge potential for digital communication methods for the rapid and widespread communication of AMR awareness materials to rural aquaculture communities in Bangladesh and across Asia. Our results support the need for more research into the most appropriate and effective content of AMR awareness campaigns for aquaculture communities and question the need for explaining the science underlying AMR in such communication materials.

## Background

The overuse of antibiotics in animals in low- and middle-income countries (LMICs) is a serious issue that is contributing to the increasing global burden of antimicrobial resistance (AMR) [1,2]. Although improving awareness and understanding of AMR is a key strategic objective of the World Health Organisation’s (WHO) global AMR action plan [3], most large-scale awareness campaigns have been focused on addressing the overuse of antibiotics in humans only and have been carried out in high-income countries, where the issues needing to be addressed can differ from those in LMICs [4,5]. Large-scale health awareness campaigns in LMICs have traditionally involved radio/TV mass media, printed materials and interpersonal communication strategies [6], which can be challenging due to the substantial associated costs [7] and geographical isolation of communities. Previous AMR awareness campaigns have also cited communicating the complexity of the social and scientific issues underpinning AMR to people with limited educational background as a barrier to successful implementation [4]. To this end, digital communication technologies offer much scope: static or animated graphics with audio voiceover can provide engaging materials for maximal accessibility, and can illustrate the underlying microscopic-scale concepts of AMR much more easily than through the use of traditional print-based material with diagrams and photos. Importantly, these digital materials can also be distributed to large audiences quickly and at low cost, through social media. Many LMICs have rapidly growing internet networks, giving access to rural communities that traditionally have been time-consuming and costly to reach.

South East Asia has been identified as the global area posing the greatest risk to AMR dissemination [8]. In this region, aquaculture is a major food production industry that supplies both domestic and international markets. The global aquaculture industry poses a significant and largely unrecognised risk to global AMR dissemination due to the widespread and indiscriminate use of antibiotics, high levels of disease, poor biosecurity and a lack of wastewater treatment, all exacerbated by the industry’s direct interconnectivity with both land and aqueous environments [9]. Thus, aquaculture communities are a key future target for international AMR awareness campaigns. In this pilot project, we sought to evaluate the scope for digital animation to rapidly and effectively communicate AMR messages to aquaculture farmers in Bangladesh, where aquaculture accounts for 3.57% of GDP, provides around half of the Bangladesh population’s dietary animal protein, and sustains the lives of 17 million people (10% of the population) [10]. There is much capacity for digital dissemination of AMR materials in Bangladesh since in recent years the Bangladesh Government has invested in creating an extensive and rapidly growing 4 G internet network: in December 2019 there were over 99 million internet subscribers (60% of the population), 94 million (57% of the population) of which accessed the internet through a mobile phone. Mobile internet usage is growing rapidly, with an average of 10 million new users per year between December 2016 and December 2019 [11].

## Methods

### Production of animation

**Figure 1.**
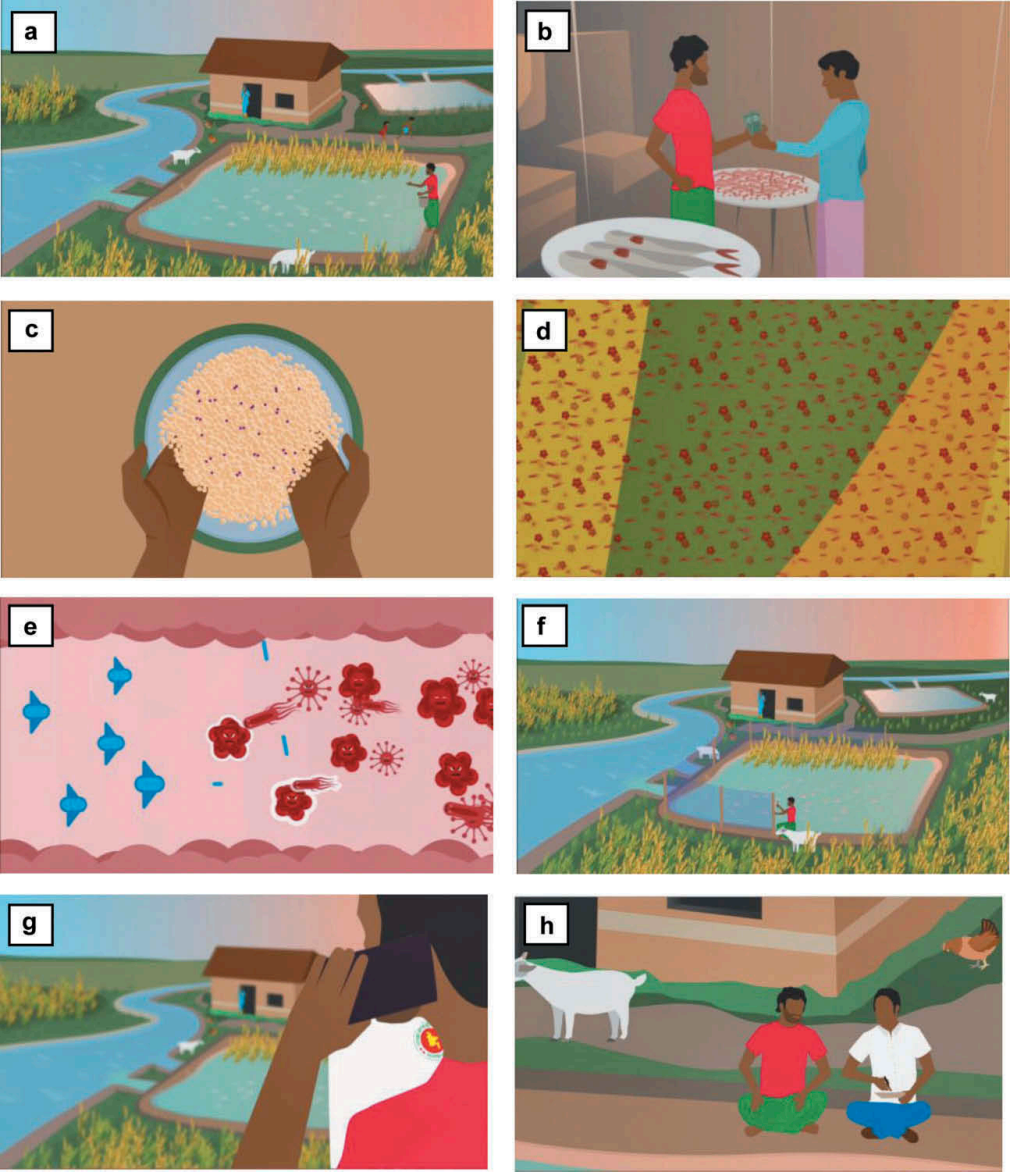
Animation design

In order to establish the most appropriate messages to convey to a Bangladesh aquaculture audience, we consulted with a range of aquaculture industry stakeholders and community engagement practitioners at an ‘AMR, One Health and Aquaculture’ workshop, held in Dhaka, Bangladesh, in February 2019 [12,13]. The resulting messages (Table 1) were then integrated into a 4-minute digital animation, whose design (landscape, characters, dress and background music) and script was developed through close collaboration between the project partners, with the aim of being as culturally relevant as possible (Figure 1). Bangla and English versions of the animation were created and can be viewed at https://www.youtube.com/watch?v=P_mnoK5E53Q (Bangla) and https://www.youtube.com/watch?v=zR0EB9P7rKc (English).

**Table 1.** Key messages and points identified through consultation with aquaculture stakeholders and community engagement practitioners

General educational messages	Community-specific messages	Key points to consider
What are bacteria?	Officers for accurate diagnosis; ask what is in any prescription, to know if it includes antibiotics	With positive messages about aquaculture farming
What are antibiotics?	Mix antibiotics with feed; do not throw them directly into the water	The animation should give economic reasons for prudent antibiotic usage in order to maximise uptake of messages
What is antibiotic resistance?	Do not use antibiotics in combination	It should be made clear that antibiotics are needed in some circumstances
Improve biosecurity to avoid disease and the need for antibiotics		For maximal understanding, the term ‘antibiotic’ should be used (not ‘antimicrobial’) and we should avoid the use of the term ‘resistance’

(a) The landscape, characters, dress and background music were designed through close collaboration between the project partners, with the aim of being as culturally relevant as possible. The community-specific messages, general messages and key points outlined in Table 1 were illustrated or explained via audio in the animation, including (b) positive messages about fish farming; (c) antibiotics should be added with feed and not used in combination; (d) the idea that bacteria are everywhere and (e) that resistant bacteria are not killed by antibiotics; (f) that practicing good pond management will keep fish healthy; and (g) to call local fisheries officers for advice on diagnosis and treatments. (h) The animation also depicts good practice on the part of local fisheries officers, in sitting and talking to farmers about the problem.

### Field testing

In May 2019 we visited 36 aquaculture farms (shrimp, fish-shrimp or fish-shrimp-vegetation) in two of the major aquaculture-producing districts of Bangladesh, Khulna (20 farmers) and Bagherat (16 farmers), to seek feedback on the animation. Before showing farmers the animation, we collected preliminary information on their behaviour, in terms of access/use of medicines and levels of awareness of AMR. The majority of people surveyed (83%) were male, and most of the farms were extensive (97%), small-medium sized (total pond area less than 20,000 m^2^; 92%), and farmed a combination of shrimp, prawn and fish species (91%) (Supplementary Information). Farmers were then shown the animation on a tablet, and feedback was collected through a questionnaire. All data were collected using the SurveyMonkey app.

### Social media campaign

On 30 May 2019, the animation was released via the WorldFish Bangladesh Facebook channels. Within Bangladesh, Facebook is the most widely used social media platform, and between this date and 5 December 2019, the Facebook page containing the Bangla version of the animation was viewed 17,614 times. On 1 June 2019, a targeted email marketing campaign press release (https://www.worldfishcenter.org/news-media/multimedia) with information on the animation and links to the English/Bangla version of the animation was also distributed to 364 people at 185 organisations, including media outlets, Bangladesh Government, aquaculture-related businesses, non-governmental organisations, donor organisations and educational institutions. An English version of the animation was posted on the WorldFish and CGIAR (WorldFish parental organisation) global twitter accounts with 97,900 impressions (the number of twitter feeds it was displayed in) and 11,200 views.

## Results

### Determining the most appropriate AMR messages to convey to aquaculture farmers

Consultation with a wide range of industry stakeholders suggested a need to include general educational messages on the science underlying bacteria, disease, antibiotics and AMR since awareness levels of these topics were believed to be generally low (Table 1). Community-specific messages were also required, including the need for better pond management and biosecurity, as well as specific messages on antibiotic application to ponds. An important discussion point raised was whether to include messages that are realistic for farmers to achieve, such as ‘mix antibiotics with feed’, versus those which are more idealistic but less feasible at the moment, such as ‘seek diagnosis before treating’, which is impractical as most farmers in Bangladesh currently have low levels of access to diagnostic facilities and advice from Fisheries Officers. Despite farmers’ limited access to Fisheries Officers, it was agreed that the animation should support the Bangladesh Government’s plans to improve this service and so farmers should be encouraged to contact Fisheries Officers; however, the animation would promote best practice amongst Fisheries Officers, for example, by depicting them taking the time to sit with farmers and discuss the issue properly before advising on treatment, rather than rushing. Consultation with community engagement practitioners also identified the need to reinforce positive general messages about aquaculture farming, to emphasise the economic benefits of prudent antibiotic usage to farmers and to make it clear that these drugs are needed in some circumstances (Table 1).

### Engaging fish farming communities with AMR messages through digital media

**Figure 2.**
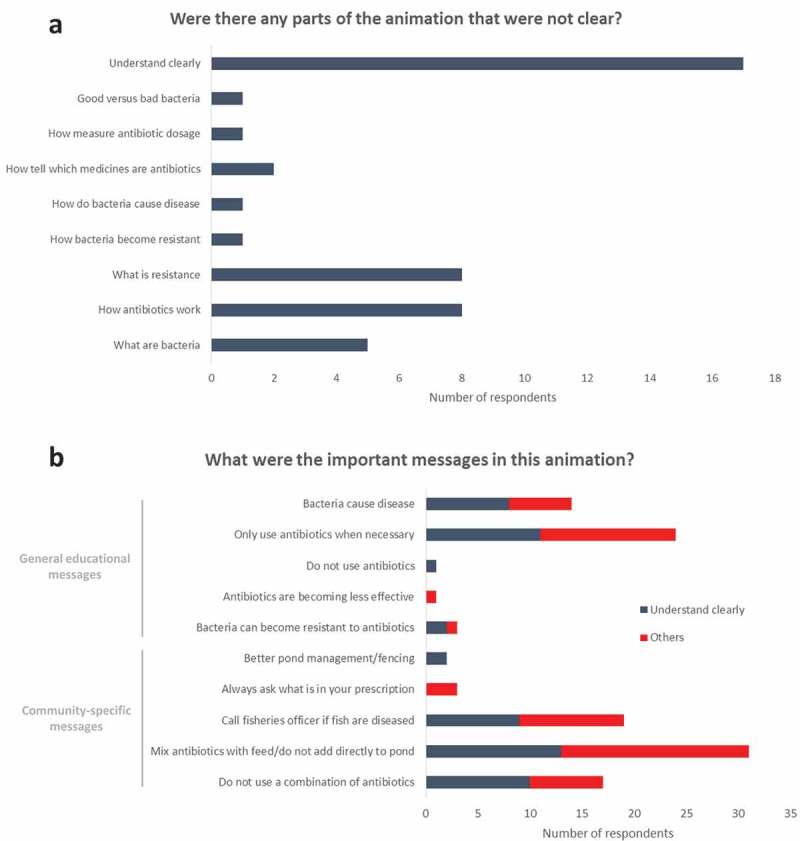
Farmer feedback on animation content

Upon field testing of the animation, every farmer stated that they thought this was a good method of communicating with them, and almost half (47%) of farmers said they understood the animation clearly (Figure 2(a)). When asked about the most important messages conveyed, farmers listed both the general educational and community-specific messages outlined in Table 1. Interestingly, the same messages were listed by both farmers who felt that they had understood the animation clearly and those who had not (Figure 2(b)). These data support the idea that digital communication tools, such as animations, can effectively convey messages on appropriate antibiotic use and AMR to aquaculture farmers, even when they claim to have not fully understood the full content.

Thirty-six aquaculture farms (shrimp, fish-shrimp or fish-shrimp-vegetation) were visited in May 2019. After being shown the animation, farmers were asked open-ended questions to collect feedback and their responses were grouped as shown. (a) Responses to ‘Were there any parts of the animation that were not clear?’. (b) Responses to ‘What were the important messages in this animation?’, categorised according to whether the farmer stated that they had understood clearly or otherwise in (a). All data were collected using the SurveyMonkey app.

Prior to being shown the animation, none of farmers claimed to have heard of ‘antibiotic resistance’ or ‘drug resistance’, and after viewing 97% said that it would change the way they use antibiotics in the future (35/36; with one ‘maybe’). We did not question whether this was an increase or decrease in usage, since it is difficult to obtain reliable information on shrimp farmers’ use of antibiotics due to the growing pressure within the industry to reduce antibiotic usage in line with export antibiotic residue checks [9]. Thus, although the majority of farmers (86%) stated that they did not know which treatments they used contained antibiotics, we cannot be certain about the accuracy of these figures. However, our data support other studies indicating that farm shops play a major role in the advice (74%) and sales (100%) of chemical treatments, including antibiotics, and therefore should be a key target to address antibiotic sales and usage in future AMR action plans [12,14].

**Figure 3.**
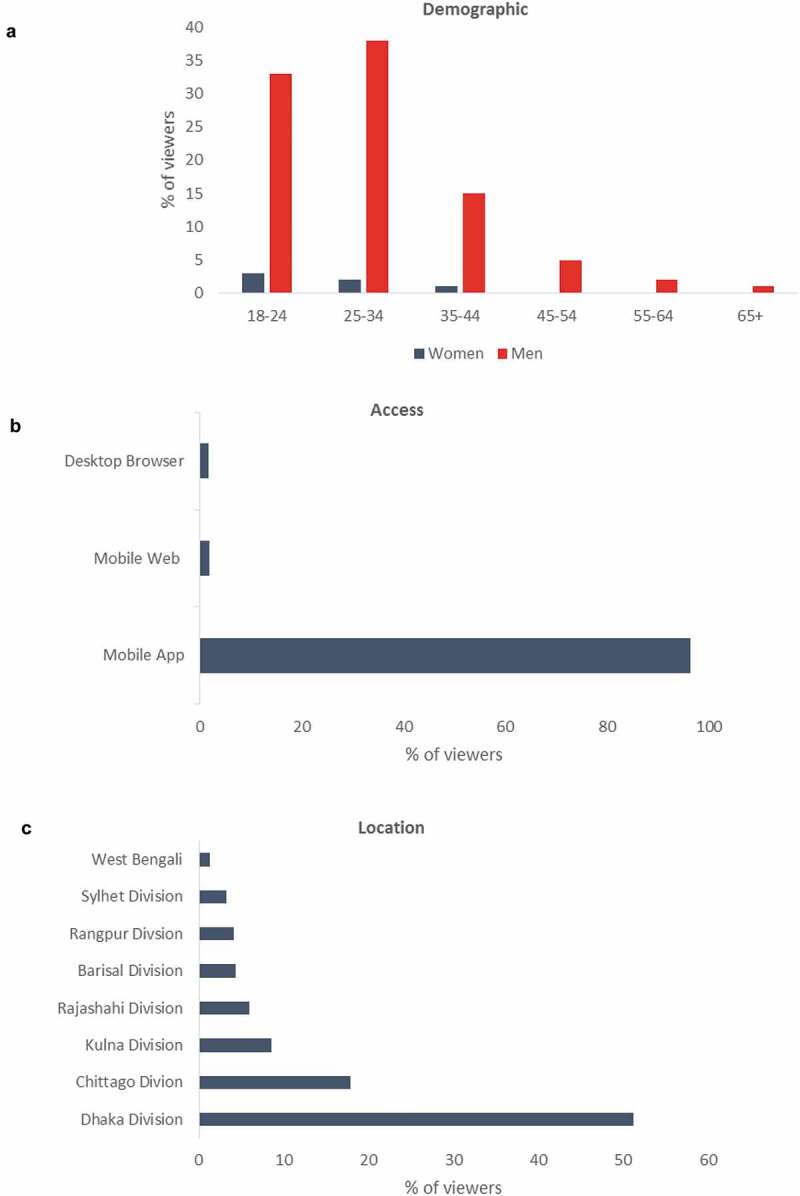
Facebook metrics from the social media campaign

Dissemination of the animation via a social media campaign resulted in 17,614 Facebook views (Bangla version) and 11,200 Twitter views (English version) in a 6-month period. Analysis of the Facebook metrics showed that the vast majority of the viewers were male (94%), under 35 (76%), and accessed the animation via a mobile app from the Dhaka division of Bangladesh (Figure 3), reflecting the fact that the majority of mobile phone owners in Bangladesh are male and the Dhaka division is the most populous [15,16]. Although these preliminary data demonstrate the potential for using social media to rapidly disseminate AMR messages to a large audience in Bangladesh, they do not provide us with information on what proportion of the people who accessed the animation were stakeholders within the Bangladesh aquaculture industry, or how much of the animation they watched. Thus, future studies should better evaluate the target audience and determine the most effective social media routes to reach these rural aquaculture communities.

Data collected between 30th May 2019 and 5th December 2019, showing (a) viewer demographic, (b) method of access and (c) location.

## Discussion

The international AMR community now acknowledges that, for maximal effect, large-scale awareness campaigns should be integrated within industry-wide AMR behavioural policy strategies [4,17]. Our preliminary data suggest that, by rapidly and effectively engaging rural aquaculture communities, digital communications could effectively support this. Despite high initial costs in creating digital materials, their dissemination is relatively low cost and low maintenance compared to traditional print-based materials. Further, digital materials can be easily edited and high levels of interest can be sustained over time by linking the digital materials to other ongoing relevant social media campaigns (e.g. public health campaigns, aquaculture industry campaigns) and progressively releasing additional materials (e.g. other AMR videos, stories, etc.) to build the campaign and increase the reach. As an example, our animation was used alongside other digital materials during AMR awareness week (18–24 November 2019), which led to an additional 1214 new viewers in those 6 days alone.

This study supports others that have shown an urgent need for more research into the most appropriate content of AMR awareness campaigns, to ensure that they achieve their intention to effect behavioural changes [18,19]. Future studies should assess to what extent these digital materials impact on behaviours associated with usage. The farmers we questioned in the field cited the same important take-home messages irrespective of whether they felt that they had understood the content (Figure 2(b)); therefore, if the level of uptake is also similar this questions whether inclusion of the complex scientific basis of AMR in awareness campaigns is necessary.

An important limitation of our study was that Facebook does not disclose information on viewer’s profiles; therefore, we could not identify whether the 17,614 views were by people within the Bangladesh aquaculture industry. This could be addressed in future studies by linking a survey to the animation or by creating a closed Facebook group made up of the WorldFish followers based in Bangladesh, which would allow us to extract user information and be more specific in our campaign targeting. If we are successful in validating social media as a viable route for communication with aquaculture communities, then the potential impact of digital communications is considerable: interactive communication materials could be used for two-way dialogue with audiences that are very difficult to otherwise reach, allowing them to engage with industry and academic stakeholders. Accessible online courses tailored to these rural communities could also offer unprecedented access to resources that have previously been restricted due to financial, social, cultural or geographic constraints, and these could easily be translated into different languages, allowing international organisations to leverage materials and better disseminate them across regions that face similar problems. This is particularly relevant for the global aquaculture industry, with 89% of production occurring in countries neighbouring Bangladesh who face similar borderless challenges from climate change, disease and environmental pollution [20].

## Conclusion

This pilot study demonstrates the potential for digital media as an effective tool for the rapid and widespread communication of AMR messages to rural Bangladesh aquaculture farmers and could be used to target other rural LMIC communities in Bangladesh and beyond, who are similarly challenging to reach. Importantly, our data also support the urgent need for more research into the most appropriate content for digital AMR awareness campaigns, since with the enormous audiences involved it is imperative to ensure that they are as effective as possible in achieving their intended outcome.

## Supplementary Material

Supplemental Material

## Paper context

Aquaculture is a major food production sector in Asia and poses significant risk to AMR dissemination; thus, aquaculture communities are key targets for AMR awareness campaigns. Our pilot study demonstrates the huge potential for digital media to rapidly and effectively communicate AMR-related messages to Bangladesh aquaculture communities. We highlight the importance of multi-stakeholder co-ordination in identifying industry-specific AMR messages, and the need for further research into the most appropriate content when developing AMR awareness campaigns.
