# Supplementary material for: Raising awareness of antimicrobial resistance in rural aquaculture practice in Bangladesh through digital communications: a pilot study
Source: Glob Health Action. 2020 Mar 10;12(Suppl):1734735. doi: 10.1080/16549716.2020.1734735 (PMC7144293; doi:10.1080/16549716.2020.1734735)
Supplement: Supplemental Material [file ZGHA_A_1734735_SM6669.docx]

**Supplementary Information: Responses to farmer survey**

Data collected during visits to 36 aquaculture farms in May 2019, prior to showing farmers the animation. All data was collected using the SurveyMonkey app.

Q1: Gender?

| Male | 30 | 83% |
| --- | --- | --- |
| Female | 6 | 17% |

Q2: What is your role on the farm?

| Farm owner | 31 | 86% |
| --- | --- | --- |
| Farm worker | 2 | 6% |
| Farmer’s wife | 3 | 8% |

Q3: District?

| Khulna | 20 | 56% |
| --- | --- | --- |
| Bagherat | 16 | 44% |

Q4: Type of farm? s=shrimp, p=prawn, c=carp, t=tilapia, h=horina, pa=paisha.

| Extensive* | 34 | 94% |
| --- | --- | --- |
| Semi-intensive* | 1 | 3% |
| Skipped | 1 | 3% |

**Extensive farming is the traditional method of aquaculture farming, with low stocking densities in larger ponds, and less stock and pond management. Semi-intensive farming involves higher stocking densities and greater pond management, such as increased water exchange, use of fertiliser, etc. Fully intensive farming is relatively uncommon in Bangladesh.*

| s + p | 3 | 8% |
| --- | --- | --- |
| s + c | 1 | 3% |
| s + p + c | 23 | 64% |
| s + p + h | 1 | 3% |
| s + p + pa | 1 | 3% |
| s + p + c + t | 4 | 11% |
| Skipped | 3 | 8% |

Q5: Size of farm (in decimals*)?

**A decimal is the traditional unit for area used in Bangladesh aquaculture. 1 decimal = 40.46m^2^*

| 0-50 | 5 | 14% |
| --- | --- | --- |
| 51-100 | 7 | 19% |
| 101-500 | 20 | 56% |
| >500 | 4 | 11% |

Q6: Who do you get advice from on which treatments to use?

| Neighbour/friend farmer | 18 | 50% |
| --- | --- | --- |
| Cluster lead | 1 | 3% |
| Farm shop | 25 | 69% |
| Department of Fisheries Office | 2 | 6% |
| NGO | 6 | 17% |
| Own experience | 1 | 3% |
| Company representative | 2 | 6% |
| Skipped | 2 | 6% |

Q7: Where do you buy your treatments from?

| Local farm shop | 36 | 100% |
| --- | --- | --- |
| Big city chemical shop | 1 | 3% |
| Sales people/agents | 2 | 6% |

Q8: Have you ever contacted your local fisheries officer?

| Yes | 7 | 19% |
| --- | --- | --- |
| No | 29 | 81% |

Q9: If yes in Q8, what advice did you ask for (n=7)?

| Advice on disease | 3 | 43% |
| --- | --- | --- |
| How to prevent disease | 3 | 43% |
| Water quality testing | 1 | 14% |
| How to remove lice | 1 | 14% |

Q10: If no in Q8, what has stopped you from doing this (n=29)?

| Got information elsewhere | 2 | 7% |
| --- | --- | --- |
| Fisheries officer is too far from farm | 18 | 62% |
| Has not been necessary | 2 | 7% |
| Fisheries officers don't respond properly | 7 | 24% |

Q11: How do you know which of your treatments are antibiotics?

| Read it on the packaging | 4 | 11% |
| --- | --- | --- |
| Shopkeeper tells them | 4 | 11% |
| Friend/other farmer tells them | 0 | 0% |
| Don’t know which ones are antibiotics | 21 | 58% |
| Don’t use antibiotics | 11 | 31% |
| Skipped | 10 | 28% |

Q12: Do you keep a record of which treatments you use?

| Yes | 0 | 0% |
| --- | --- | --- |
| No | 36 | 100% |

Q13: Have you heard of antibiotic resistance/drug resistance?

| Yes | 0 | 0% |
| --- | --- | --- |
| No | 35 | 97% |
| Skipped | 1 | 3% |
